# Supplementary material for: Splice-Junction-Based Mapping of Alternative Isoforms in the Human Proteome
Source: Cell Rep. Author manuscript; Available in PMC 2020 Jan 15. (PMC6961840; doi:10.1016/j.celrep.2019.11.026)
Supplement: 2 [file NIHMS1546469-supplement-2.zip › DF1/PXD006675/MitralValve/MitralValve_4_TNXB_MGPLSVVIVTVAR.pdf]

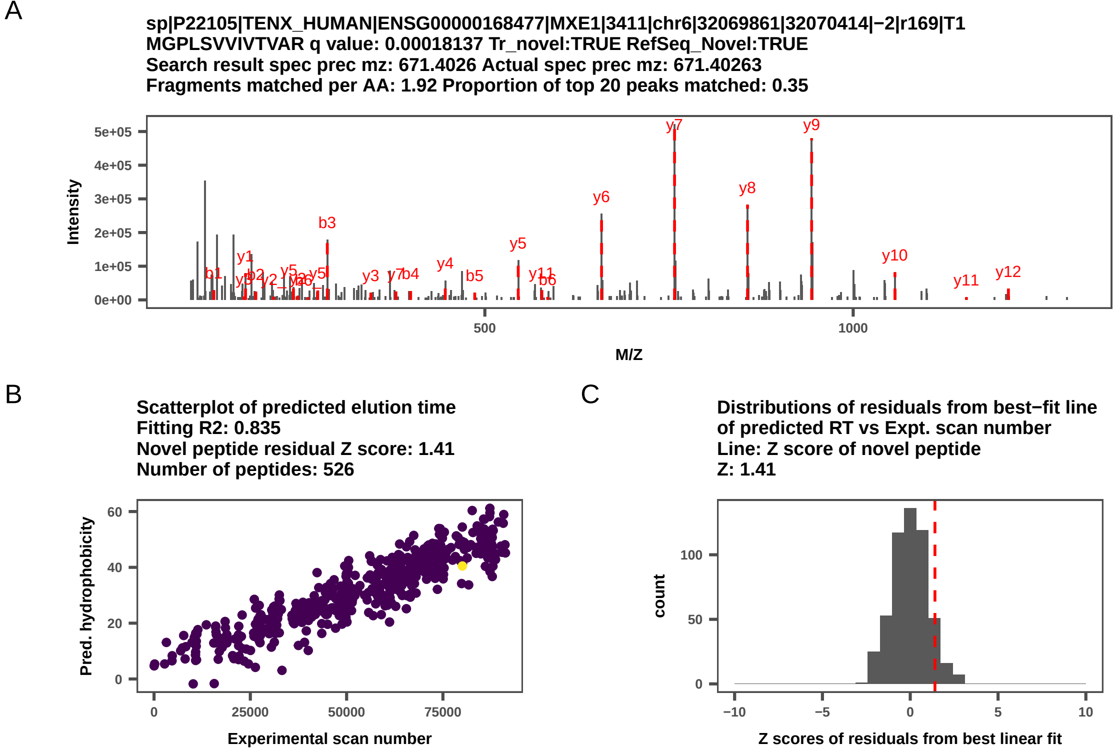
A
sp|P22105|TENX_HUMAN|ENSG00000168477|MXE1|3411|chr6|32069861|32070414|−2|r169|T1
MGPLSVVIVTVAR q value: 0.00018137 Tr_novel:TRUE RefSeq_Novel:TRUE
Search result spec prec mz: 671.4026 Actual spec prec mz: 671.40263
Fragments matched per AA: 1.92 Proportion of top 20 peaks matched: 0.35
Intensity
5e+05
4e+05
3e+05
2e+05
1e+05
0e+00
y7
y9
y8
y6
b3
y5
y10
y4
b5
y1
b1
y3
b4
y11
b6
y11
y12
500
1000
M/Z
B
Scatterplot of predicted elution time
Fitting R2: 0.835
Novel peptide residual Z score: 1.41
Number of peptides: 526
Pred. hydrophobicity
60
40
20
0
0
25000
50000
75000
Experimental scan number
C
Distributions of residuals from best−fit line
of predicted RT vs Expt. scan number
Line: Z score of novel peptide
Z: 1.41
count
100
50
0
−10
−5
0
5
10
Z scores of residuals from best linear fit
